# Favorable Impact on Stress-Related Behaviors by Modulating Plasma Butyrylcholinesterase

**DOI:** 10.1007/s10571-017-0523-z

**Published:** 2017-07-15

**Authors:** Stephen Brimijoin, Susannah Tye

**Affiliations:** Mayo Clinic, Rochester, MN USA

**Keywords:** Butyrylcholinesterase, Ghrelin, Anxiety, Stress disorders, Aggression, Mouse models, Viral gene transfer, Long term reduction of stress hormone

## Abstract

In the last decade, it has become clear that the neuropeptide “ghrelin” and its principal receptor have a large impact on anxiety and stress. Our recent studies have uncovered a link between plasma butyrylcholinesterase (BChE) and ghrelin. BChE actually turns out to be the key regulator of this peptide. This article reviews our recent work on manipulating ghrelin levels in mouse blood and brain by long term elevation of BChE, leading to sustained decrease of ghrelin. That effect in turn was found to reduce stress-induced aggression in group caged mice. Positive consequences were fewer bite wounds and longer survival times. No adverse effects were observed. Further exploration may pave the way for BChE-based treatment of anxiety in humans.

## Introduction

The present era is rife with anxiety and stress across the globe. In modern cultures, stress is pervasive and nearly inescapable. When paired with individual biological and/or psychological vulnerability, chronic stress can lead to serious mental and physical health issues that impact broad populations (Grippo and Johnson 2009; Puterman et al. 2016; Walker et al. 2014). These issues include heightened risk of emotional disorders and their progression across the lifespan (Puterman et al. 2016; Walker et al. 2014). Bolstering resilience to stress should be one of our best options for preventing and treating the significant health consequences of this epidemic. Richard Kvetnansky was a pioneer researcher in this arena for many years. At the NIH in the 1970s, using mice and rats, he provided solid evidence that repeated stress from prolonged restraint leads to chronically elevated levels of hormones in the adrenocortical axis. These hormones included norepinephrine, epinephrine, adrenocorticotropic releasing hormone (ACTH), and corticosterone (the rodent homologue of cortisol) (Gewirtz et al. 1971; Kvetnansky et al. 1995). Such effects are now well-established as having a substantial impact on physical and mental health, as repeatedly shown over the last four decades in Kvetnansky’s laboratory and those of increasingly many investigators worldwide.

A relatively new approach to prevention and treatment of anxiety disorders is based on the concept that stress-resilience is anchored in the promotion of biological resilience to stress at the cellular level (Puterman et al. 2016; Walker et al. 2014; Yamanaka et al. 2016). In part, this is achieved by enhancing metabolic functions essential to maintain adequate levels of usable energy in the brain (Bayliss and Andrews 2013; Packer et al. 1997). In other words, metabolic capacity and efficiency are emerging as keys to prevent stress-induced dysfunction in the brain circuitry that governs emotions and related phenomena (Walker et al. 2014). As yet, the specific factors that differentiate vulnerable from resilient human subjects are still far from understood. However, it is clear that, from mouse to man, individuals who are particularly vulnerable to the deleterious health effects of stress are likely to react by expressing persistent responses of “learned helplessness” (King et al. 1993). That is, passive, helpless, or pessimistic responses to stress have been shown to exacerbate and extend stressful states once they are triggered (Petty et al. 1997; Zhukov and Vinogradova 2002). In addition to impairing an individual’s ability to cope effectively with the stressor, such a behavioral response triggers a sequelae of biological events that further exacerbate the stress response and associated processes including inflammation and mitochondrial impairment that in turn impact neural circuit function, particularly in regions regulating mood and anxiety (Enkel et al. 2010; Petty and Sherman 1982). Over time, chronic stress exposure has been shown to accelerate cellular aging as demonstrated by reduced telomerase activity and telomere length. In contrast, resilient individuals demonstrate much more robust ability to cope actively with stressors at both the behavioral and the physiological levels (Oliveira et al. 2016). Recent works from our two laboratories and others suggest that cellular resilience to metabolic stress is crucial for buffering the behavioral and physiological impacts of emotional stress (Tye 2013; Tye et al. 2014). What is more, both of these phenomena are strongly affected by the peptide hormone “ghrelin,” originally regarded as just a “hunger hormone”.

### Roles for Ghrelin in Emotional States

The intersection of basal metabolism with emotional balance is plainly evident in the manifold and widespread roles of ghrelin. This acylated octapeptide is produced primarily in gastric tissue and released into the blood stream, but it is also generated by specific neuronal populations in the hypothalamus and selected other brain regions (Delporte 2013; Kojima et al. 1999). Phasic increases of circulating ghrelin stimulate gastric muscle contractions colloquially referred to as “hunger pangs.” More or less simultaneously with gastric effects, ghrelin activates vagal afferent terminals leading directly from the stomach to stimulate brain areas involved in appetite and food seeking (Delhanty et al. 2013).

Numerous recent studies also implicate ghrelin in behaviors and states that have no obvious connection to nourishment. In particular, it has become apparent that ghrelin directly affects anxiety, stress, and fear-related behaviors by virtue of its actions on “GHSR,” the growth hormone secretagogue receptor (Harmatz et al. 2017). This fact became clear to us as we followed long-term, group-housed, sibling male mice to determine the safety of interventions for sustaining elevated plasma levels of butyrylcholinesterase (BChE) to treat cocaine abuse (Chen et al. 2015). These animals unexpectedly showed a sharp drop in spontaneous aggression. Computer searches for stress-related hormones with ester groups that BChE might hydrolyze led to a report by Chuang and Zigman (2010) focusing on the enzyme and ghrelin. We soon found that our mice with BChE overexpression had substantially lower plasma ghrelin than did the controls. Such an outcome, though focused on fighting behaviors, was plausibly related to a reduction in social anxiety that would ordinarily have promoted inter-sibling attacks (Chen et al. 2015). Continued research along those lines soon led us to conclude that BChE, long considered a nonspecific drug metabolizer, has a true physiological role in ghrelin inactivation and is, essentially, a ghrelin hydrolase. As such, BChE may serve as a useful agent for direct modulation of stress-related physiological and behavioral responses.

Given ghrelin’s multiplicity of effects it is unsurprising that there has been much confusion regarding the precise nature of the interactions with its target, the growth hormone secretagogue receptor, GHSR1a. One reason for the confusion is the unusual behavior of that receptor. Unlike many others, GHSR1a typically exist in a partially active and tonically desensitized state, even in the absence of agonist. Early on, this peculiar behavior led to confusion among researchers in the field. In particular, there was uncertainty as to whether ghrelin was a GHSR agonist or antagonist. We initially assumed that ghrelin was a stress-producer, given that untreated mice with normal plasma ghrelin were fighting much more than the BChE-treated mice with *low* plasma ghrelin. Abundant new evidence, from our lab and others (Chen et al. 2015; Harmatz et al. 2017; Lutter et al. 2008; Meyer et al. 2014) indicates that the opposite is true.

What appears to resolve the superficially contradictory evidence at hand is that there are two different processes involving ghrelin’s actions in the brain. Thus, ghrelin release from the stomach rises and falls in waves that coincide with increased appetite and food seeking, but the range of peptide concentrations in the blood is not great, and the rate of change is not rapid. In contrast, ghrelinergic neurons that synapse on feeding centers in brain provide rapid spikes of ghrelin to act on postsynaptic receptors. Such an arrangement means that synaptic release of ghrelin should be more effective on brain receptors in animals or humans with *low levels* of the circulating peptide. That is, low tonic ghrelin levels in brain should confer increased postsynaptic receptor sensitivity to transient, phasic fluxes of ghrelin in the brain. In contrast, high levels of circulating ghrelin will weaken ghrelin signaling at brain synapses, owing to reduced sensitivity or expression of the GHSR1a receptor. The implication of the impact of ghrelin tone on receptor sensitivity is of great importance given that the apparent mission of those receptors is to release growth hormone in the pituitary and other brain regions which in turn function as antianxiety, antifear, antistress, and antiaggression agents (Harmatz et al. 2017).

**Fig. 1:**
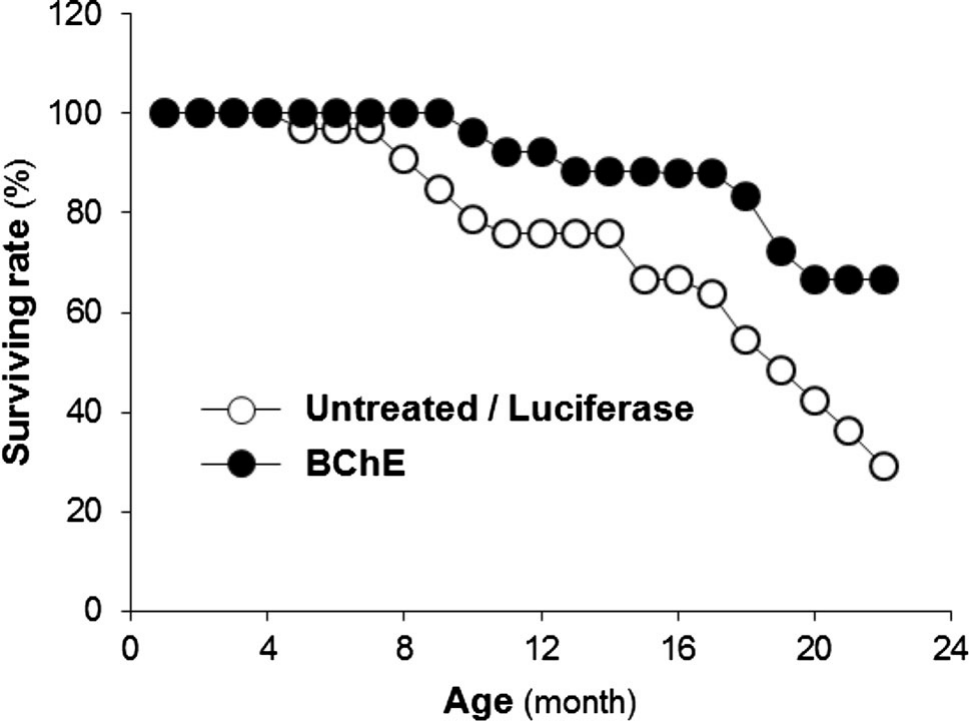
Unpublished findings from long-term daily observations of group-housed male mice (32 pooled controls and 30 mice transduced at 6 weeks with adeno-associated virus (AAV) or helper-dependent virus vector encoding mutated mouse BChE: “mBChE mut”). Plasma BChE levels in the vector-treated groups were ~100-fold above normal and were sustained for the following 2 years. Initially, mice were housed five per cage, until signs of fighting arose (4–5 months), when they moved to single-cage housing. The varied housing conditions prevent a definitive judgment of true lifespan although ANOVA showed a significant main effect of treatment (controls vs. pooled vector-treated groups): *F*_1.60_ = 13.64, *p* < 0.001). No post hoc testing was performed

**Fig. 2:**
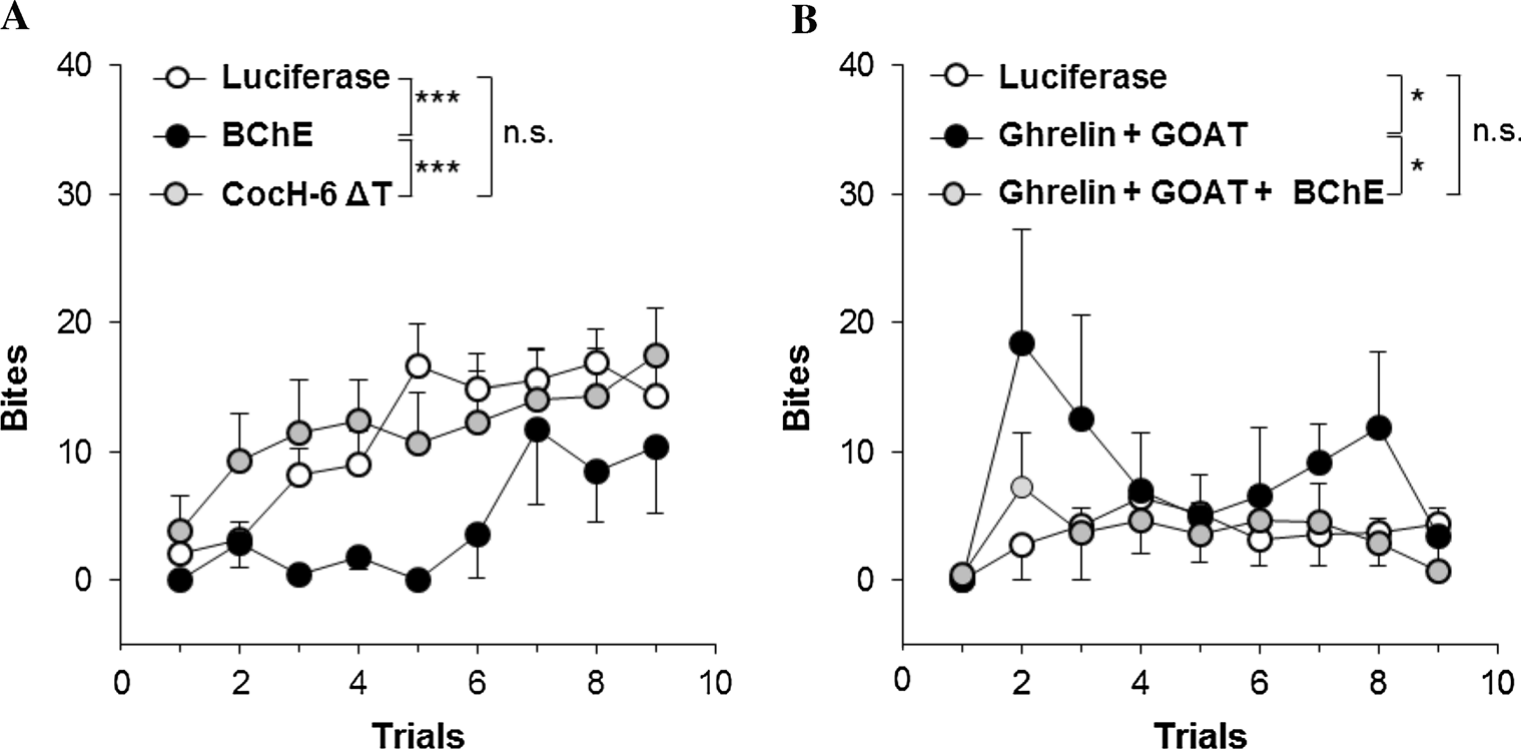
Bite scores in confrontations between a resident male mouse and a male intruder (Chen et al. 2015). **a** 3-month Balb/c with AAV-luciferase vector (*n* = 18) versus mBChE mutant vector-treated (*n* = 7); AAV-CocH-6 ΔT (human BChE E1-V529 with A199S/F227A/S287G/A328 W/Y332G/E441D)-treated mice (*n* = 14). **b** AAV-luciferase-treated 3-month C57BL/6 wild type (*n* = 9) versus same age C57BL/6 treated simultaneously with AAV vectors encoding cDNA for ghrelin and ghrelin octanoyl acyl transferase (GOAT) (*n* = 6); and same-age mice treated triply, with vectors for ghrelin, GOAT, and mBChE mutant (*n* = 9). *p < 0.05; ***p < 0.001; *n.s.* not significant

We serendipitously developed a model based on an AAV viral vector that reduces circulating ghrelin for the life of a mouse by driving large and sustained elevations of BChE (Schopfer et al. 2015). This approach provides an important new tool for modulating circulating ghrelin levels. Specifically, our research has shown that long-term lowering of plasma ghrelin by BChE bolsters physiologic resilience (increased health and longevity (Fig. 1) and reduces social stress-related behaviors (reduced spontaneous fighting and associated injuries (Fig. 2) and (Brimijoin et al. 2016; Chen et al. 2015). We hypothesize that altered stress reactivity is key to these observations. If so, then a stable reduction of “background ghrelin” should increase central cellular responses to ghrelin to bolster stress resilience and alleviate anxiety and depression-like behaviors. This hypothesis can easily be tested by manipulating ghrelin levels up or down with viral gene transfer or by utilizing a range of gene knockouts to reach similar outcomes. Complementing this, it also remains possible that our findings can be explained, at least in part, via an increase in the efficiency of ghrelin-mediated signaling. That is, a stable reduction of ghrelin may increase constitutive signaling through the ghrelin receptor as a direct result of an increase in membrane presence of GHSR1a, relative to when levels of ghrelin are high. Indeed, it may be that this constitutive signaling is particularly important for the antistress/antianxiety effects.

#### Ghrelin Signaling and Neuroprotection/Proliferation

It is already clear that ghrelin is a mediator of cellular metabolic capacity, and that it plays a key role in bolstering cellular resilience by increasing mitochondrial function and regulating cell proliferation, apoptosis and inflammation-related signaling pathways in a GHSR-1a dependent manner (Chung et al. 2007). GHSR-1a signaling may play an important role in maintaining stress resilience by protecting cellular integrity and buffering the potentially deleterious effects of chronic HPA-axis activation. We suspect that the neuroprotective effects of GHSR-1a can be enhanced by long term modulation of ghrelin tone. In our view, clarifying ghrelin’s neuroprotective mechanisms would be a good step toward developing novel prevention and treatment strategies.

Ghrelin stimulates release of growth hormone by activating GHSR1a, the growth hormone secretagogue receptor (Howard et al. 1996) and has multiple nonendocrine functions that serve to regulate neuronal behavior (Chung et al. 2013). Consequently, ghrelin signaling impacts diverse brain functions, including learning and memory (Diano et al. 2006), reward, and motivation (Naleid et al. 2005, Abizaid et al. 2006, Jiang et al. 2006), stress-responsivity and mood (Carlini et al. 2004, Lutter et al. 2008), as well as neuroprotection and neurogenesis (Jiang et al. 2006, 2008, Chung et al. 2007, 2013, Miao et al. 2007, Hwang et al. 2009, Moon et al. 2009a, b, Lee et al. 2010a, b).

Ghrelin can boost cellular resilience, increase mitochondrial function, and reduce apoptosis after ischemic insult in hippocampal, cortical or hypothalamic neurons, either in vivo or in vitro (Bali and Jaggi 2016; Bayliss and Andrews 2013, 2016; Diano et al., 2006). This occurs through direct, GHSR1a-dependent, modulation of mitogen-activated kinase (MAPK) and extracellular-signal-regulated kinase (ERK1/2) signaling (Bayliss and Andrews 2013). Ghrelin has also been shown to increase the proliferation of cultured hippocampal neural stem cells in a GHSR1a-dependent manner (Chung et al. 2013). Receptor levels rose substantially after treatment and rapid activation of MAPK, ERK1/2, and PI3 k/Akt pathways. Modulation of downstream effectors, such as glycogen synthase kinase (GSK)-3b and mammalian target of rapamycin (mTOR), were also observed. However, pretreatment with specific inhibitors of MAPK, ERK1/2, PI3 K/Akt, and mTOR attenuated ghrelin-induced cell proliferation (Chung et al. 2013).

#### Ghrelin Signaling and Behavioral Resilience

Chronic stress and its long-term physiological and psychological sequelae are harmful to both brain and body. The mechanism by which stress induces adaptations in the neural circuitry to impair behavioral resilience has recently been elucidated to a certain degree. The molecular pathways involved in neuroprotection and glial proliferation bolster not only resilience against damaging effects on neurons and supporting cells. They also enhance *behavioral* stress resilience, by facilitating ‘active coping’ responses (Duman and Voleti 2012). There is mounting evidence that ghrelin, via actions at GHSR1, plays an important role in regulating stress-responsivity—although the direction of effect remains unclear. Systemic and central administration of ghrelin has been shown to induce anxiety-like behavior in mice (Asakawa 2001; Carlini 2002) by activating the hypothalamic–pituitary–adrenal axis (Zigman et al. 2006). However, anxiolytic effects of ghrelin have also been reported. Thus, increased ghrelin levels (achieved through calorie restriction or direct peptide injection) demonstrably *decrease* anxiety and depressive-like behaviors in a GHSR1a-dependent fashion (Lutter 2008). The reasons for the disparate responses are unclear. In light of these conflicting data, together with development of new tools for modulating ghrelin tone, we are placing a high priority on further research into stress-related phenomena and the potential roles of BChE and ghrelin in exacerbating or reducing the impact of stress disorders. The potential to buffer stress effects at the cellular, systems, and behavioral levels now seems to have real therapeutic potential, warranting full investigation and rapid clinical translation.
